# Comparison of transforming growth factor beta expression in healthy and diseased human tendon

**DOI:** 10.1186/s13075-016-0947-8

**Published:** 2016-02-17

**Authors:** Henry C. J. Goodier, Andrew J. Carr, Sarah J. B. Snelling, Lucy Roche, Kim Wheway, Bridget Watkins, Stephanie G. Dakin

**Affiliations:** Botnar Research Centre, Nuffield Department of Orthopaedics, Rheumatology and Musculoskeletal Sciences, University of Oxford, Nuffield Orthopaedic Centre, Windmill Road, Headington, OX3 7LD UK; NIHR Oxford Biomedical Research Unit, Botnar Research Centre, University of Oxford, Windmill Road, Oxford, OX3 7LD UK

**Keywords:** Tendon, Tendinopathy, Fibrosis, TGFβ

## Abstract

**Background:**

Diseased tendons are characterised by fibrotic scar tissue, which adversely affects tendon structure and function and increases the likelihood of re-injury. The mechanisms and expression profiles of fibrosis in diseased tendon is understudied compared to pulmonary and renal tissues, where transforming growth factor (TGF)β and its associated superfamily are known to be key drivers of fibrosis and modulate extracellular matrix homeostasis. We hypothesised that differential expression of TGFβ superfamily members would exist between samples of human rotator cuff tendons with established disease compared to healthy control tendons.

**Methods:**

Healthy and diseased rotator cuff tendons were collected from patients presenting to an orthopaedic referral centre. Diseased tendinopathic (intact) and healthy rotator cuff tendons were collected via ultrasound-guided biopsy and torn tendons were collected during routine surgical debridement. Immunohistochemistry and quantitative real-time polymerase chain reaction were used to investigate the protein and gene expression profiles of TGFβ superfamily members in these healthy and diseased tendons.

**Results:**

TGFβ superfamily members were dysregulated in diseased compared to healthy tendons. Specifically, TGFβ-1, TGFβ receptor (R)1 and TGFβ R2 proteins were reduced (*p* < 0.01) in diseased compared to healthy tendons. At the mRNA level, *TGFβ R1* was significantly reduced in samples of diseased tendons, whereas *TGFβ R2* was increased (*p* < 0.01). *BMP-2, BMP-7* and *CTGF* mRNA remained unchanged with tendon disease.

**Conclusions:**

We propose that downregulation of TGFβ pathways in established tendon disease may be a protective response to limit disease-associated fibrosis. The disruption of the TGFβ axis with disease suggests associated downstream pathways may be important for maintaining healthy tendon homeostasis. The findings from our study suggest that patients with established tendon disease would be unlikely to benefit from therapeutic TGFβ blockade, which has been investigated as a treatment strategy in several animal models. Future studies should investigate the expression profile of fibrotic mediators in earlier stages of tendon disease to improve understanding of the targetable mechanisms underpinning tendon fibrosis.

## Background

Musculoskeletal diseases are a common cause of pain and disability in well-resourced health systems [1]. Shoulder pain is the third most common cause of musculoskeletal pain, accounting for 2.4 % of all general practitioner consultations annually in the UK [2]. Shoulder pain is frequently treated conservatively, but after 1 year 41 % of patients have persistent pain [3]. Rotator cuff disease is the major cause of shoulder pain with the supraspinatus tendon being most frequently affected [4]. The onset of tendon pathology is associated with ageing, chronic overuse and genetic predisposition [5–7]. Shoulder pain is a major socio-economic burden and joint failure from rotator cuff tears can lead to secondary osteoarthritis [8].

Patients with rotator cuff disease may have tendinopathy (whereby the tendon is structurally intact) or a tendon tear. Tendon tears are sub-classified according to tear sizes ranging from small (<1 cm) to massive (>5 cm in length) [9]. Tendons heal by fibrosis and the scar repair is frequently structurally and functionally inferior to normal tendon, increasing the risk of re-injury [10–13]. Treatment for shoulder tendinopathy includes physiotherapy, non-steroidal anti-inflammatory drugs (NSAIDs), and interventional treatments including local infiltration with glucocorticoids, platelet-rich plasma (PRP) and arthroscopic acromioplasty or rotator cuff repair. Non-operative strategies are associated with mixed patient outcomes [14]. Furthermore, surgical repairs of torn rotator cuff tendons are associated with high failure rates of up to 94 % [15].

Inflammation is known to drive fibrotic repair in skin, liver, renal and pulmonary diseases. Signal transducer and activator of transcription (STAT)-6 pathways encompassing interleukin (IL)-13/IL-4 are thought to be the dominant pro-fibrotic axis for increasing collagen synthesis from fibroblasts and regulating alternatively activated macrophages [16–19]. Upregulation of IL-13 has been shown to mediate tissue fibrosis via transforming growth factor (TGF)β [20]. TGFβ is the most frequently investigated effector of fibrosis and regulator of extracellular matrix (ECM) turnover. The TGFβ superfamily modulates a number of vital cellular processes in tissue development, differentiation and homeostasis. TGFβ-1 is pivotal to wound healing processes and implicated in multiple fibrotic disease states including connective tissue fibrosis [21]. TGFβ is synthesised as a pro-peptide, and is abundant within the ECM, platelets and macrophages. Signalling is mediated through binding of active TGFβ to a heteromeric complex of TGFβ receptor (R)1 and 2, resulting in SMAD3 phosphorylation and activation of target genes. Macrophages, lymphocytes and resident stromal cells are key cell populations implicated in the propagation of fibrotic disease. Whilst inflammation has been identified in diseased tendons [22, 23], little is known about the inflammatory mechanisms driving tendon fibrosis.

Animal models have been used to identify potential treatment strategies for fibrotic diseases. These include manipulation of pro-fibrotic pathways through exogenous anti-TGFβ, antisense-TGFβ gene, SMAD3 knockout, and macrophage depletion models [24–29]. Reducing TGFβ signalling diminishes the fibrotic phenotype of tendon but also reduces tissue tensile strength [30]. As part of the TGFβ superfamily, bone morphogenic proteins (BMPs) have previously been investigated for their role in tendon-to-bone healing [27, 31, 32] and in the stimulation of rotator cuff tendon-derived cells in tissue culture models [33]. These studies show BMP-2 and -7 induce collagen production in rotator cuff tendon-derived cells, suggesting these BMPs could play a role in the development of tendon fibrosis.

Little is known of the mechanisms underpinning fibrosis in diseased human tendons. To our knowledge, no study to date has investigated TGFβ superfamily expression in diseased human tendons from living patients. The aim of this study was to investigate mRNA and protein expression of key TGFβ superfamily members in tissue samples from patients with established tendon disease compared to healthy control tendons. We hypothesised that expression of TGFβ superfamily genes and proteins would be dysregulated in diseased compared to healthy tendons.

## Methods

### Diagnosis of rotator cuff tendon disease in patient cohorts

Patients presenting to a referral shoulder clinic had failed non-operative treatment, including a course of physical therapy, and glucocorticoid injections into the sub-acromial space, and had experienced pain for a minimum of 6 months. Patients had not received glucocorticoid or PRP for 12 weeks before surgery. A shoulder specialist diagnosed sub-acromial impingement syndrome in all patients. Impingement tests were positive in all patients. High-definition ultrasound examination was performed in all patients to determine if there was evidence of a rotator cuff tear and also to identify abnormal echogenic changes at the supraspinatus footprint. Exclusion criteria for all patients in this study included previous shoulder surgery, dual shoulder pathological lesions, significant problems in the other shoulder, rheumatoid arthritis or systemic inflammatory disease, osteoarthritis or significant neck problems.

### Patient cohort for gene expression analysis

Torn rotator cuff (supraspinatus, n = 7) and healthy hamstring (n = 7) tendon tissue samples were collected to investigate mRNA expression of TGFβ superfamily members in these samples. Healthy hamstring tendons were collected from patients undergoing anterior cruciate ligament (ACL) reconstruction surgery from male and female patients aged between 22 and 49 (mean 31 ± 9.5) years. Torn supraspinatus tendon tissues were collected from male and female patients aged 40 to 67 (mean 52.5 ± 9) years (Table 1). Fresh tissue samples were immediately snap frozen in liquid nitrogen and stored at –80 °C prior to RNA extraction.

**Table 1:** Patient demographics for the study group

Cohort	n	Median age (years)	Sex (M:F)	History	Treatment	Median OSS
Healthy supraspinatus	10	24 (18–29)	9:1	Shoulder instability	Shoulder stabilisation	N/A
Healthy hamstring (mRNA)	7	31 (22–49)	3:4	ACL rupture	ACL repair	N/A
Tendinopathic supraspinatus	23	54 (36–73)	13:10	Shoulder Sx 1–10 years (mean 2.5 years)	1–2 steroid injections, SAD ± PRP	29 (11–38)
Torn supraspinatus	30	69.5 (50–78)	18:12	5 small	Rotator cuff repair	20.5 (7–35)
				9 medium		
				9 large		
				7 massive		
Torn supraspinatus (mRNA)	7	52.5 (40–67)	5:2	3 medium	Rotator cuff repair	26 (9–31)
				2 large		
				2 massive		

*ACL* anterior cruciate ligament, *F* female, *M* male, *N/A* not available, *OSS* Oxford shoulder score, *PRP* platelet-rich plasma, *SAD* sub-acromial decompression surgery

### RNA extraction and cDNA synthesis

Frozen tissue samples stored at –80 °C were homogenised in 1 mL RNABee (AMS Biotechnology, UK) using an IKA Ultra Turrax T8 Homogeniser (Fischer Scientific, UK). RNA extraction was carried out as per the manufacturer’s protocol using an RNeasy mini kit (Qiagen, Limburg, Netherlands) with an on-column DNA treatment using DNase 1 (Thermo Scientific, UK). RNA concentration and quality were determined by measuring the ratio of absorbance at 260:280 nm using a NanoDrop 100 spectrophotometer (Thermo Scientific, MA, USA), with all samples achieving a minimum ratio of 1.80. RNA (250 ng) was reverse transcribed using a High Capacity Reverse Transcription Kit (4368813, Applied Biosystems, UK).

### Gene expression by quantitative real-time polymerase chain reaction

cDNA was diluted to 2.5 ng/μL with RNase free water and 5 μL was used in a 20 μL quantitative polymerase chain reaction (qPCR) with Fast SYBR Green Master Mix (4385612, Applied Biosystems). Validated human primers included *TGFB1* (QT00000728), *TGFBR1* (QT00083412), *TGFBR2* (QT00014350), *BMP2* (QT00012544), *BMP7* (QT00068936), and *CTGF* (QT00052899) (Qiagen). Duplicate reactions for each gene were run on a ViiA7 qPCR machine (Applied Biosystems, CA, USA) and the mean value for these duplicates calculated and used for analysis. Results were calculated using the ΔΔC_t_ method and normalized against β-actin and GAPDH reference genes. Results were consistent with both reference genes and data shown are normalized to β-actin.

### Patient cohort for immunohistochemistry

For this controlled laboratory study, torn supraspinatus tendons were collected at the time of surgery from the edges of torn tendons from symptomatic male and female patients with full thickness tears aged between 50 and 78 years (Table 1). Samples of intact tendinopathic supraspinatus were collected from male and female patients at the time of arthroscopic acromioplasty. All patients had loss of shoulder function and/or shoulder pain as reflected in a median Oxford shoulder score (OSS) of 29 [34]. Healthy supraspinatus tendon samples were collected from patients who underwent surgery for shoulder instability and who had an intact non-degenerative supraspinatus tendon on ultrasound confirmed at surgery. Healthy patients were aged between 18 and 29 years (Table 1). For patients undergoing general anaesthesia for shoulder stabilization or arthroscopic acromioplasty, a biopsy was taken using ultrasound guidance whilst the patient was anaesthetized and prior to the surgical procedure. A percutaneous ultrasound-guided biopsy technique was performed as previously described [35] to acquire healthy tissue 5–10 mm posterior to the anterior edge of the tendon.

### Tissue processing

Samples were immersed in 10 % buffered formalin for 1 week. After fixation, tendons were processed using a Leica ASP300S tissue processor and embedded in paraffin wax. Tissues were sectioned at 4 μm using a rotary microtome (Leica Microsystems Ltd, UK) and collected onto adhesive glass slides and baked at 60 °C for 30 minutes and 37 °C for 60 minutes.

For antigen retrieval, slides were baked at 60 °C for 60 minutes and combined deparaffinization and antigen retrieval was performed by submerging slides in FLEX TRS antigen retrieval fluid using a PT Link machine (Dako, Glostrup, Denmark). Immunostaining was performed using an Autostainer Link 48 machine using the EnVision FLEX visualisation system (Dako). Primary antibodies against TGFβ family mediators included TGFβ-1 (ab64715), TGFβ R1 (ab31013) and TGFβ R2 (ab78419) (Abcam Cambridge, UK). Antibody binding was visualized using FLEX 3,3′-diaminobenzidine (DAB) substrate working solution and haematoxylin counterstain (Dako) as per protocols provided by the manufacturer. Isotype control antibodies included mouse IgG_1_, IgG_2a_, IgG_2b_, IgG_3_ and IgM, (Dako). All antibodies were validated in-house to ensure the recommended concentration produced positive staining with minimal artifact on supraspinatus tendon tissue. After staining, slides were rehydrated in alcohols and xylene and mounted using Pertex mounting medium (Histolab, Gothenburg, Sweden).

### Immunofluorescence for co-localization of TGFβ with macrophage markers

Immunofluorescence staining of sections of massive supraspinatus tendon tears is described in detail elsewhere [22]. Briefly, after antigen retrieval, tissues were blocked in 5 % goat serum (Sigma) at room temperature. Sections were incubated with the primary antibody cocktail (macrophage markers CD206 ab117644, CD163 LS_C174770 and pan TGFβ ab66043) diluted in 5 % normal goat serum in phosphate-buffered saline (PBS) for 2 hours. Slides were washed in PBS with Tween and incubated in the secondary antibody cocktail (goat anti-mouse FITC IgG_1_ (Southern Biotech), goat anti-mouse IgG_2a_ Alexa Fluor 568, and goat anti-rabbit IgG Alexa Fluor 633 (Life Technologies)) diluted in 5 % normal equine serum (Sigma) for 2 hours. After washing, sections were incubated in 2 μM POPO-1 nuclear counterstain (Life Technologies) diluted in PBS containing 0.05 % Saponin (Sigma). Tissue autofluorescence was quenched with a solution of 0.1 % Sudan Black B (Applichem). Slides were mounted using fluorescent mounting medium (VectaShield), sealed and stored at 4 °C until image acquisition. For negative controls the primary antibody was substituted for universal isotype control antibodies: cocktail of mouse IgG_1_, IgG_2a_, IgG_2_b, IgG_3_ and IgM (Dako) and rabbit immunoglobulin fraction of serum from non-immunised rabbits, solid phase absorbed (Dako). Stained slides were visualised on a Zeiss LSM 710 confocal microscope as previously described [22].

### Image analysis for quantitative immunohistochemistry

Twenty images of immunostained sections (or until exhausted) were taken on a Zeiss inverted microscope (Zeiss, Cambridge, UK) using Axiovision software (Zeiss) at ×100 magnification with oil immersion. Images were collected systematically to ensure no overlap and to avoid areas of artefact or fold in the tissue sample. ImageJ (National Institutes of Health, Bethesda, Maryland, USA) was used to analyse the images, employing a previously validated algorithm that quantifies DAB staining by a colour deconvolution method [36, 37]. The quantification system used compared the number of nuclei to the amount of immunopositive staining to account for tissue cellularity.

### Statistical analysis

Statistical analysis was performed using GraphPad Prism 6 (GraphPad, CA, USA). Normality was tested using a Shapiro-Wilk test. Kruskal-Wallis tests were used to compare expression of TGFβ superfamily at mRNA and protein levels in samples of healthy, tendinopathic and torn tendons. *p* < 0.05 was considered statistically significant.

### Study approval

Ethical approval for this study was granted by the local research ethics committee: Oxfordshire REC B refs: 10/H0402/24, 10/H0605/35, 09/H0605/111, 09/H0606/11. Full informed consent according to the Declaration of Helsinki was obtained from all patients.

## Results

### Diseased tendons show dysregulation of TGFβ family mediators at the mRNA level

**Fig. 1:**
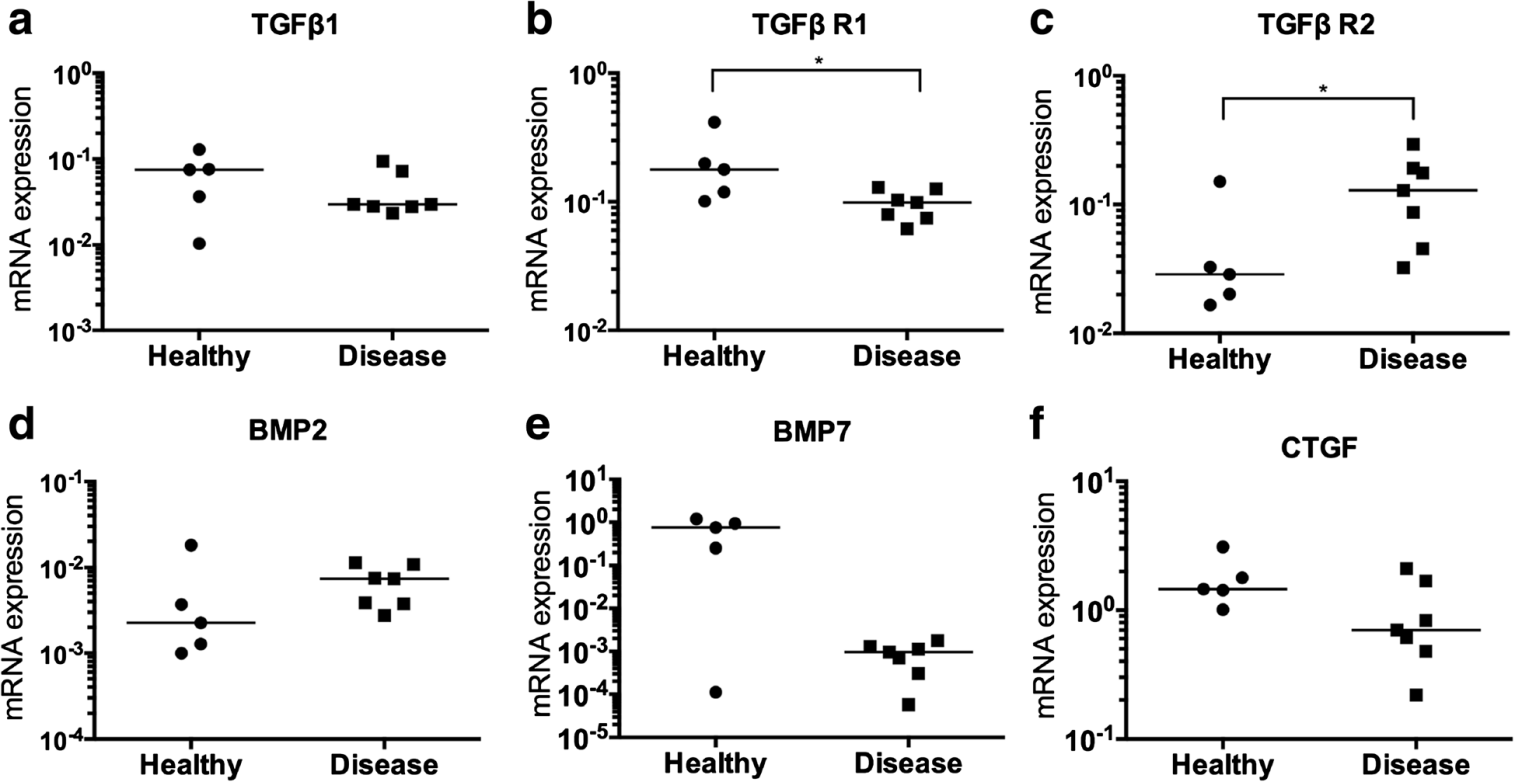
Expression of pro-fibrotic genes in healthy and diseased tendons. **a**–**c** TGFβ family mediators, **d** BMP-2 and **e** BMP-7, and **f** CTGF mRNA expression are shown in healthy hamstring (n = 7) compared to diseased rotator cuff tendons (n = 7). Gene expression is normalized to β-actin; bar shows median value. Data were analysed using the Kruskal-Wallis test; *p < 0.05. *BMP* bone morphogenic protein, *CTGF* connective tissue growth factor, *TGF* transforming growth factor, *TGF R* transforming growth factor receptor

Expression of *TGFB1, TGFBR1*, and *TGFBR2* genes were investigated in torn rotator cuff and healthy hamstring tendons. *TGFBR1* mRNA was decreased in diseased rotator cuff compared to healthy tendons (*p* = 0.048, twofold); in contrast, *TGFBR2* was increased (*p* = 0.048, threefold) (Fig. 1). There was a trend for reduced *TGFB1* in diseased compared to healthy hamstring tendons. BMPs have been shown to signal via homodimeric receptors from the TGFβ superfamily, and are also implicated in fibrotic pathways. There was no significant difference in *BMP2* mRNA between healthy hamstring and torn rotator cuff tendons (Fig. 1). *BMP7* was not detected in torn rotator cuff tendons; however, this was not significantly different compared with healthy hamstring. Connective tissue growth factor (CTGF) was also investigated as a pro-fibrotic cytokine and downstream effector of TGFβ. There was a trend for reduced *CTGF* in torn rotator cuff compared to healthy hamstring tendons.

### Histological assessment of diseased rotator cuff tendons

Tendon tissues from patients with supraspinatus tendinopathy or tears showed marked disorganization of the tendon ECM and collagen fibrils and increased vascularity. Histological assessment of tendons collected from this cohort was performed on haematoxylin and eosin stained sections using the Bonar scoring system (0–12) evaluating tissue structure, and is reported in a previous published study [22]. Median and interquartile ranges of healthy supraspinatus tendons exhibited more normal tissue architecture (Bonar score of 2, range 1–2) compared to tendinopathic (7, range 6–8) and torn supraspinatus tendons (10, range 8.25–10).

### Diseased tendons show downregulation of TGFβ superfamily proteins

**Fig. 2:**
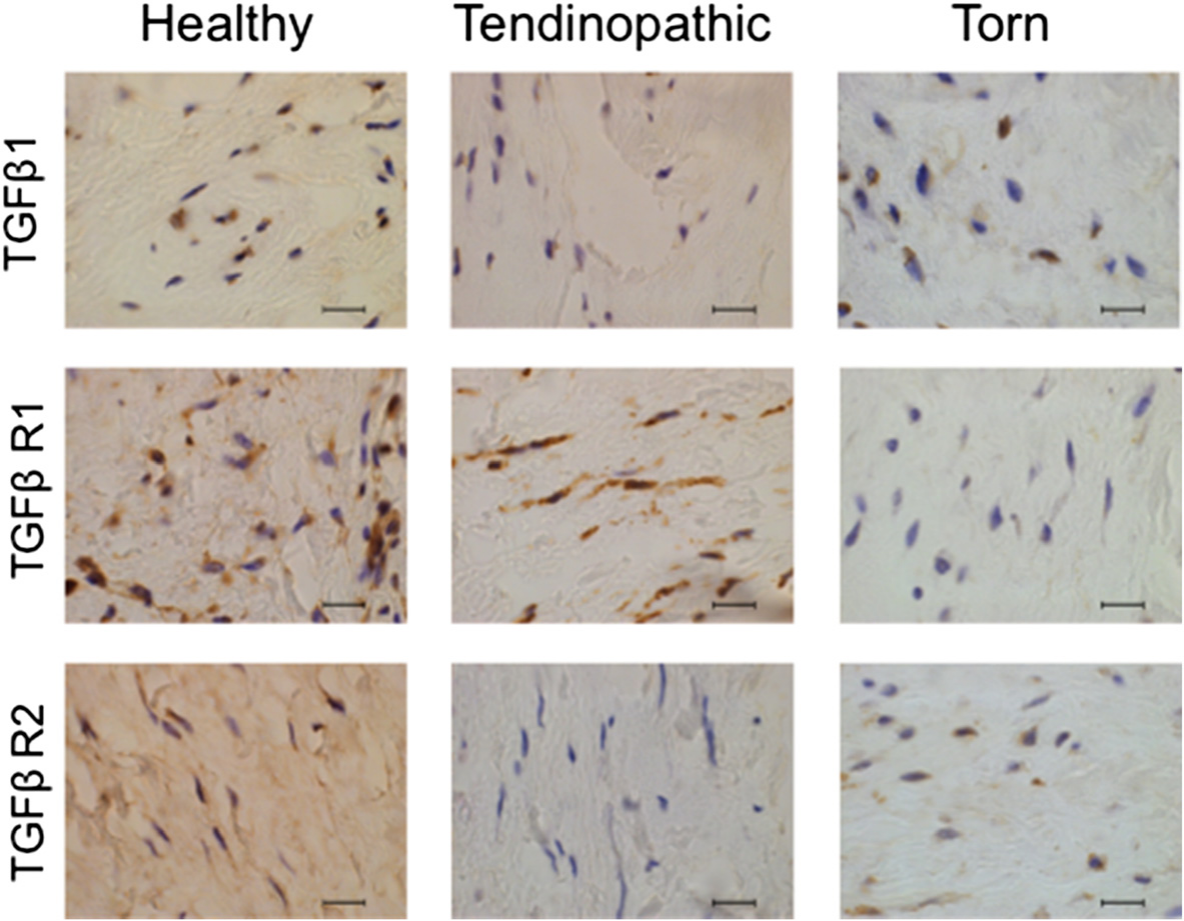
Photomicrographs showing immunostaining for TGFβ1, TGFβ R1 and TGFβ R2 in healthy, tendinopathic and torn rotator cuff tendons. Representative images of DAB immunostaining (*brown*) are shown. Nuclear counterstain (*blue*) is haematoxylin. Scale bar = 20 μm. *TGF* transforming growth factor, *TGF R* transforming growth factor receptor

**Fig. 3:**
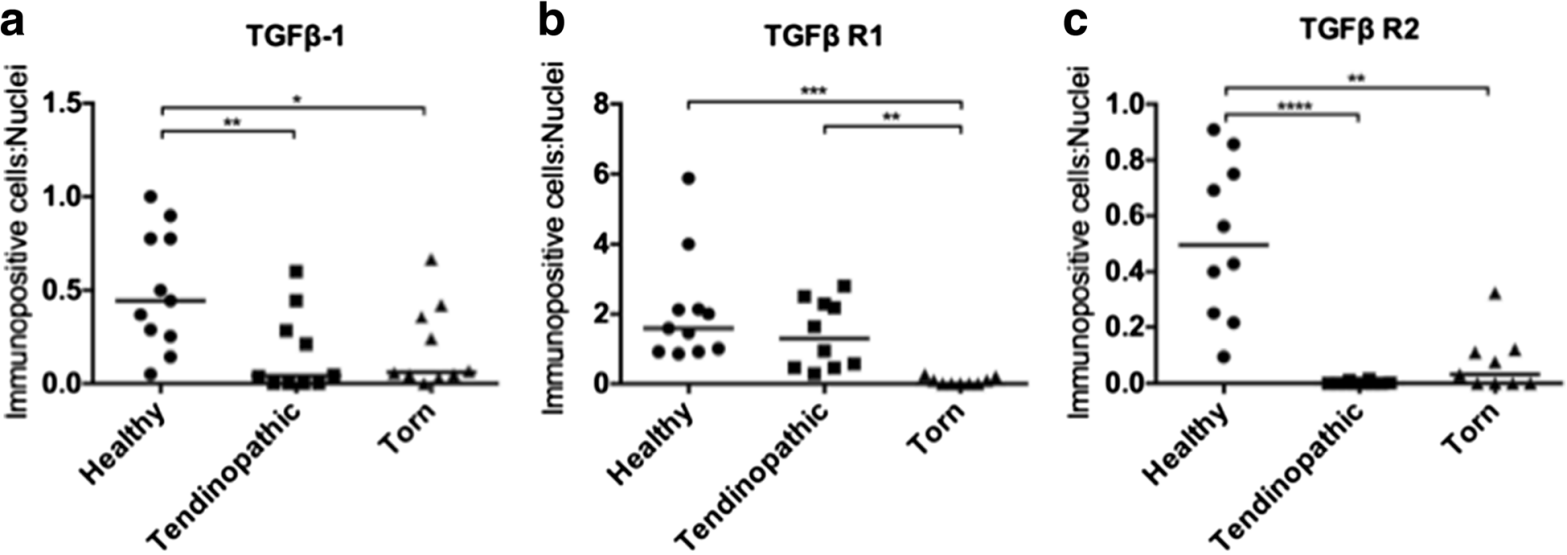
Quantitative analysis of immunopositive staining showing dysregulation of TGFβ family proteins in diseased compared to healthy rotator cuff tendons. **a** TGFβ-1: healthy (n = 11), tendinopathic (n = 10), torn (n = 10); **b** TGFβ R1: healthy (n = 10), tendinopathic (n = 10), torn (n = 9); and **c** TGFβ R2: healthy (n = 10), tendinopathic (n = 10), torn (n = 9). Bar represents the median value. Data were analysed using the Kruskal-Wallis test; *p < 0.05 **p < 0.01 ***p < 0.001 ****p < 0.0001. *TGF* transforming growth factor, *TGF R* transforming growth factor receptor

**Fig. 4:**
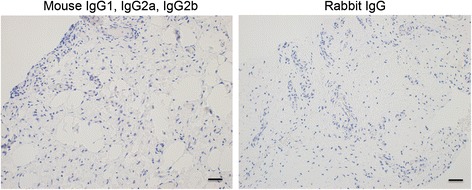
Isotype control staining of diseased human rotator cuff tendons. Panel shows representative images of diseased tendon sections stained with isotype control antibodies for mouse IgG1, IgG2a, IgG2b and rabbit IgG fractions. Nuclear counterstain is haematoxylin. Scale bar = 50 μm. *Ig* immunoglobulin

Expression of TGFβ-1, TGFβ R1, and TGFβ R2 proteins was investigated in healthy, tendinopathic and torn rotator cuff tendons (Fig. 2). Immunopositive staining for TGFβ superfamily proteins was identified in angiofibroblastic and ECM regions of tendons. Quantitative analyses of immunopositive staining showed expression of TGFβ-1, TGFβ R1, and TGFβ R2 proteins were significantly reduced in diseased tendons compared to healthy samples (Fig. 3). Specifically, TGFβ-1 protein was reduced in torn (*p* = 0.0123, 2.6-fold) and tendinopathic (*p* = 0.0064, threefold) compared to healthy tendons. TGFβ R1 protein was reduced in torn compared to healthy and tendinopathic tendons (*p* = 0.0002, 30-fold and *p* = 0.0018, 21-fold, respectively). TGFβ R2 was reduced in torn compared to healthy tendons (*p* = 0.0087, sevenfold reduction); tendinopathic tissues showed almost no expression of TGFβ R2 compared to healthy tendons (*p* < 0.00001, 183-fold reduction). Isotype control staining of diseased rotator cuff tendons is shown in Fig. 4.

### Macrophages in diseased tendons express TGFβ

**Fig. 5:**
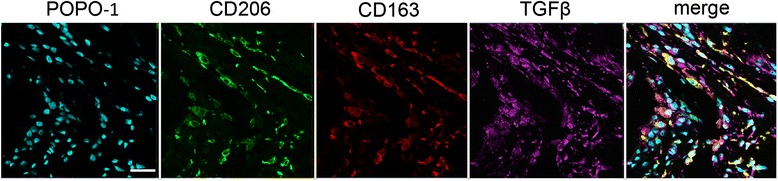
Representative immunofluorescence images of a massive supraspinatus tendon tear stained for macrophage activation markers including those in the STAT-6 pathway (CD206, *green*), the glucocorticoid receptor pathway (CD163, *red*) with pan-TGFβ (*purple*). *Cyan* represents POPO-1 nuclear counterstain. Scale bar = 20 μm. *CD* cluster of differentiation, *TGF* transforming growth factor

Macrophages are known to be important immune cell populations in diseased rotator cuff tendons and their activation status has been shown to change with disease stage [22]. We investigated if myeloid cells in samples of chronic fibrosed tendon tears expressed pan-TGFβ. Antibody labelling with macrophage markers CD206 and CD163 (representing STAT-6 and glucocorticoid receptor macrophage activation pathways, respectively) and pan-TGFβ showed co-localization of these three markers in sections of a massive supraspinatus tendon tear (Fig. 5).

## Discussion

This study of diseased tendons from well-phenotyped patient cohorts investigates expression of the TGFβ superfamily members in healthy, tendinopathic, and torn tendons. Compared to healthy tendons, diseased tendons showed dysregulation of TGFβ superfamily members. We identify suppression of TGF-β1 and TGFβ R1 mRNA and proteins in diseased compared to healthy tendons. Conversely *TGFBR2* mRNA was increased in diseased tendons. This disruption of the TGFβ axis with tendon disease suggests these pathways may have important roles in tendon homeostasis.

The progression from rotator cuff tendinopathy to tear represents a continuum. Patients with tendon tears have reduced likelihood of repair [13]. We found no difference in symptom duration between patients with intact and torn tendinopathic tendons suggesting that pain is not always associated with tendon disease. Throughout the disease spectrum, the role of TGFβ superfamily mediators and the mechanisms underpinning tendon fibrosis remain understudied. Knowledge of these pathways is better described in fibrotic diseases of other connective tissues including pulmonary and renal tissues where increased levels of TGFβ and subsequent collagen production are reported in animal and human tissues [38–40]. In these tissues TGFβ-1 is known to modulate the inflammatory response by influencing fibroblast and macrophage recruitment, stimulating collagen production and downregulating proteinase activity [18, 41]. TGFβ has been shown to regulate epithelial–mesenchymal transition (EMT), potentially generating another source of collagen-producing cells [42]. In tendon, TGFβ is stored in the ECM and released in response to exercise and strain, regulating collagen synthesis and acting as a mechanical transducer [43, 44]. Sakai et al. showed an increase in total TGFβ protein when comparing tissue from patients with rotator cuff tendon tears and anterior shoulder instability [45]. In contrast, Fenwick et al. found no evidence of TGFβ-1 protein in chronic tendinopathy or normal cadaveric human tendon tissues [46]. Chang et al stimulated and inhibited TGFβ-induced collagen production in a surgical animal model to investigate flexor tendon adhesions demonstrating the importance of TGFβ in fibrotic healing [24].

In this study, we show that TGFβ-1, TGFβ R1 and TGFβ R2 protein levels are reduced in diseased compared to healthy rotator cuff tendons. We propose this could be a protective response to limit the hypertrophic fibrosis characteristic of tendon disease. Healthy rotator cuff tendons showed a variable range of expression of TGFβ receptors and ligands, perhaps demonstrating more active regulation of tissue homeostasis by TGFβ in healthy compared to diseased tendons. This suggests an important homeostatic function similar to those proposed in skin, cartilage and vascular tissue where dysregulation can result in disease [47–51]. It is conceivable that the downregulation of TGFβ superfamily proteins in established disease could further reduce the ability of the tendon to heal. This may be exacerbated in torn tendons by their reduced ability to transmit strain, which would then suppress the mechanical stimulus to regulate TGFβ signalling [43]. Fibrotic pathways independent of TGFβ, such as tumour necrosis factor alpha, may be activated resulting in them becoming a more dominant driving force in chronic tendon disease [52].

TGFβ mRNA and protein have been shown to increase in animal models of acute-stage tendon healing. However, these models of surgical tendon transection may not accurately recapitulate chronic human tendon pathology [53, 54]. The tendon samples used in the current study represent chronic-stage tendon disease. We speculate that expression of TGFβ family mediators in human rotator cuff tendons would be increased during acute-stage pathology, and then decline in chronic disease as reported in the current study. Future work should investigate the temporal regulation of members of the TGFβ superfamily and of alternative fibrotic pathways including Wnt, PI3K and vascular endothelial growth factor in patients with early through to established tendon disease.

Although we demonstrate downregulation of TGFβ signalling mediators with established tendon disease, *TGFBR2* mRNA was upregulated in tendon tears. This observation may represent a potential aberration in the translation of *TGFBR2* mRNA into protein. Alternatively, upregulation of *TGFBR2* could be an attempt to support higher levels of alternatively activated macrophages [55]. We identified CD206^high^ and CD163^high^ macrophages that co-expressed pan-TGFβ in a massive tendon tear, suggesting M2 macrophages may be a source of TGFβ in advanced stage tendon disease.

TGFβ pathways have been investigated as potential therapeutic targets to modulate fibrosis, adhesions, and hypertrophic scarring [24, 26, 28, 30]. However, anti-TGFβ models also resulted in reduced tissue tensile strength [30, 56]. In a model of stress-shielding, exogenous TGFβ helped maintain tendon strength, whereas anti-TGFβ accelerated the loss of strength [56]. These studies and our results support the concept that TGFβ has a fundamental role in tendon homeostasis as well as playing a role in fibrosis.

CTGF is an effector cytokine of TGFβ that increases deposition of ECM and promotes myofibroblast differentiation. With significantly reduced TGFβ in tendon disease, it might be expected that CTGF would also be decreased. However, in the current study no significant difference was seen in *CTGF* mRNA expression between healthy and diseased tendons. It is possible that other signalling pathways could influence *CTGF* mRNA expression. It has been suggested that CTGF can be independently expressed via the IL-13 pathway [57] which is implicated in alternative activation of macrophages. This is an interesting observation in light of the high levels of monocytes and macrophages observed in chronic rotator cuff tendinopathy [22].

BMPs are a sub-group of the TGFβ superfamily and are known to play developmental and homeostatic functions in most tissues [58]. BMP-2 and -7 have been shown to increase collagen production in rotator cuff cells, and could both be implicated in a fibrotic phenotype [33]. Furthermore BMP-2 may, like TGFβ, be increased in response to strain [59]. However, we found no significant difference in mRNA expression of *BMP2* or *BMP7* in healthy and diseased tendons.

There are several limitations to this study. We did not investigate expression of other isoforms of TGFβ including TGFβ-2 and TGFβ-3 or expression of phospho-SMAD signalling. The samples from the current study were obtained from patients with established tendon disease. It is necessary to ascertain the temporal nature of TGFβ signalling in the more acute stage of tendinopathy and early stages of fibrosis. However, acquiring these samples of early-stage human tendon disease presents a number of challenges. We acknowledge there are limitations with the use of the hamstring tendon as a comparator to diseased tendons including tendon type and age differences. However, hamstring tendon was taken from live healthy donors with no history of tendon disease. We believe this is a more suitable comparator than cadaveric rotator cuff tendon tissues where little is known about whether the tendons were healthy or diseased and the influence of postmortem change.

The findings from our study showing downregulation of TGFβ superfamily members in established disease suggest that these patients are unlikely to benefit from therapeutic TGFβ blockade, which has been investigated as a treatment strategy in several animal models. Improved understanding of fibrotic processes in diseased human tendons is essential to inform therapeutic target discovery. Future studies should focus on investigating the expression profiles and mechanisms of fibrosis in tendon tissues obtained from longitudinal patient cohorts, from early to advanced disease and both before and after treatment.

## Conclusions

We demonstrate dysregulation of the TGFβ axis in chronic human rotator cuff tendon disease and propose this may be a protective mechanism to further limit fibrosis. Our findings also suggest TGFβ pathways may have an important role in tendon homeostasis. Further work is needed to investigate TGFβ-related pathways in healthy and diseased tendons, their roles in early-stage tendon pathology and the contribution of alternative pathways in the development of tendon fibrosis.

## Abbreviations

ACL: Anterior cruciate ligament
BMP: Bone morphogenic protein
CD: Cluster of differentiation
CTGF: Connective tissue growth factor
DAB: 3,3′-Diaminobenzidine
DNA: Deoxyribonucleic acid
ECM: Extracellular matrix
EMT: Epithelial–mesenchymal transition
Ig: Immunoglobulin
IL: Interleukin
NSAID: Non-steroidal anti-inflammatory drug
OSS: Oxford shoulder score
PBS: Phosphate-buffered saline
PRP: Platelet-rich plasma
qPCR: Quantitative polymerase chain reaction
RNA: Ribonucleic acid
STAT: Signal transducer and activator of transcription
TGF: Transforming growth factor
TGF R: Transforming growth factor receptor
